# Greening Family Medicine clinic operations and clinical care, *where do we start?* A scoping review of toolkits and aids

**DOI:** 10.1093/fampra/cmad006

**Published:** 2023-02-02

**Authors:** Sonja C Wicklum, Kate Nuique, Martina A Kelly, Colleen C Nesbitt, Jessica J Zhang, Clark P Svrcek

**Affiliations:** Department of Family Medicine, University of Calgary, Cumming School of Medicine, Calgary, AB, Canada; Department of Family Medicine, University of Calgary, Cumming School of Medicine, Calgary, AB, Canada; Department of Family Medicine, University of Calgary, Cumming School of Medicine, Calgary, AB, Canada; Department of Family Medicine, University of Calgary, Cumming School of Medicine, Calgary, AB, Canada; Department of Family Medicine, University of Calgary, Cumming School of Medicine, Calgary, AB, Canada; Department of Family Medicine, University of Calgary, Cumming School of Medicine, Calgary, AB, Canada

**Keywords:** carbon footprint, climate change, environmental health, family physician, implementation toolkit, primary healthcare

## Abstract

**Background:**

There is a pressing need for healthcare to respond to the climate crisis. Family physicians, given their central role in community healthcare provision, are strategically placed to lead, support, and promote sustainable healthcare, yet guidance on how to do this is fragmented.

**Objective:**

To identify and evaluate toolkits and aids on sustainable healthcare to act as a curated resource for family physicians and their care teams interested in delivering evidence-based sustainable healthcare in their clinical practices.

**Methods:**

A scoping review was completed of the published and grey literature across 4 databases and 2 search engines to identify articles and aids/toolkits from 1990 to present. Toolkits were subsequently evaluated for purpose, evidence-base, implementation process, adaptability to family medicine, and outcome measures.

**Results:**

The search identified 17,751 articles. Screening resulted in 20 published articles and 11 toolkits. Most articles presented simple checklists to support greening clinic initiatives, 3 studies focussed on partial carbon footprint analyses, and 4 on educational initiatives. Toolkits ranged in sustainability topics and degree of depth covered, and adaptability and outcome measures. None of the resources identified have been formally evaluated for effectiveness.

**Conclusions:**

A range of aids exist to support greening of clinic operations; however, there is a significant gap in the literature for greening clinical care. Two toolkits were found to be comprehensive, one requiring tracking and reporting of sustainability initiatives. This scoping review provides a starting point for motivated family doctors and community clinics to initiate change and support more sustainable healthcare.

**Key messages**

- The climate crisis is a healthcare crisis.
- Family physicians can “green” both clinical care and clinic operations.
- Family physicians need resources to respond to climate calls to action.
- A wide spectrum of clinic “greening” aids and toolkits exist.
- These clinic-directed sustainability aids and toolkits are in relative infancy.
- Opportunities exist for implementation and evaluation of these curated resources.

## Introduction

The climate crisis is a healthcare crisis. Left unabated, worsening environmental health will increase major diseases including cardiovascular disease, respiratory disease, cancer, mental illness, and suffering imposed by extreme weather events.^1–4^ The healthcare sector plays a vital role in mitigating the deleterious impact of rising greenhouse gases. Many healthcare organizations are committing to green policies, setting targets to support environmentally sustainable healthcare.^5–8^ Family physicians, given their central role in community healthcare provision, are strategically placed to lead, support, and promote sustainable healthcare. However, guidance on how to do this is fragmented and busy family physicians, while supportive of this culture shift in theory, are often unclear how to implement change in their clinical practice. A collated evidence-based resource to guide practice change could empower family physicians to implement sustainable planetary health practices at a community level, with cumulative national and global impact.

Healthcare contributes globally to greenhouse gas production, an estimated 4%–7% of total national emissions in developed countries.^1–3^ Eminent professional healthcare organizations have called for action, including the World Health Organisation, the American Medical Association, the Canadian Association of Physicians for the Environment, and the National Health System, United Kingdom.^5,8,9^ The specific role of primary care is widely advocated in supporting sustainable healthcare practices, endorsed by family medicine societies including WONCA (Global Family Doctors)^5^ and multiple associations from Canada, Australia, New Zealand, Scotland, and the United Kingdom.^7,8,10,11^ Organizations recognize the central position of family physicians to support planetary health directly in clinical patient care and indirectly through operations, procedures, and processes in their clinics. In clinical care, e.g. family physicians can avoid prescribing medications which contribute to greenhouse gas generation and advise on lifestyles that support “co-benefits” for patient and planet such as plant-based diets. As small businesses, family physicians can green their clinical operations by adopting circular economic procurement practices and sourcing locally where possible.

There is a willingness by family physicians to respond to these calls to action and integrate change by “greening” their practice through clinic operations or patient care. Several grassroots organizations have developed aids to assist community-based family doctors (general practitioners) in improving their clinic operations to reduce their environmental footprint and support changes in clinical care that prioritize the health of the patient while acknowledging and prioritizing the health of the planet.^9,12^ The aids that have been developed range from solely educational materials and commentaries to very advanced, multilayered, intervention strategies with corresponding tools to support the strategies. To date, as far as we are aware, these aids have not been collated or curated. Family doctors, challenged by time and resources across most countries, need aids readily available.

The purpose of this study was to identify and evaluate toolkits and aids on sustainable healthcare to act as a resource for family physicians interested in delivering evidence-based sustainable healthcare in their clinical practices.

## Methods

Our study consisted of 2 steps: first, we conducted a scoping review^13,14^ of published and grey literature to identify toolkits and aids, and secondly, we evaluated the toolkits identified.

Scoping reviews support an exploration of the breadth and depth of the literature and identify knowledge gaps for a specific subject.^15^ The structure of the scoping review allowed for investigation of the breadth of the literature, to include aids, singular projects, tools, toolkits, or educational interventions in family medicine that could be considered for replication by a family doctor and their clinical team, to integrate clinic greening policies in their setting. This review follows the Preferred Reporting Items for Systematic Reviews and Meta-analysis Protocols—extension for Scoping Reviews.^16^ The protocol is available on the Open Science Framework, osf.io/xp4t3.

### Inclusion and exclusion criteria

We established a priori criteria for inclusion and exclusion of published studies that address environmental mitigation through clinic operations and clinical care in family medicine (Table 1). We defined “clinic operations” as processes involved in the actual running of a clinic, such as energy use, procurement of equipment, recycling and waste handling, and utility use. “Clinical care” referred to the interaction between doctor and patient, the assessment and management of health concerns. “Toolkits” were defined as collections of adaptable documents to inform and facilitate implementation of evidence-based clinical interventions,^17^ in contrast to articles describing a single aid, such as a checklist or single intervention.

**Table 1.** Inclusion and exclusion criteria.

Inclusion criteria	
1	- Research studies—published articles, abstracts, and thesis - Articles guiding practice changes that are in the format of an aid (singular element such as checklist, tool, educational intervention, project) or toolkits (multiple elements)
2	- Research studies or articles focussed on family doctors (general practitioners) or clinic managers in primary care settings in community
3	- Describes clinic operations or clinical practice, including “clinic management,” “clinical care” subjects
4	- Describes how the changes will mitigate environmental degradation, including terms “adaptation,” “greening,” “climate change,” “climate smart,” “environmental impact”
5	- Any location
6	- Any journal
7	- English language articles and abstracts only
8	- Published 1 January 1990 to 30 June 2022 - 1990 selected because the first climate change assessment was published at this time by the Intergovernmental Panel on Climate Change (IPCC), a United Nations body examining the science on climate change^18^
1	- Commentaries without a tool
2	- Research studies or articles focussed on hospital operations or hospital-based practice
3	- Areas of study relevant to environmental degradation mitigation but with their own extensive literature base: (i) Deprescribing (ii) Antibiotic stewardship—or “deprescribing” of antibiotics (iii) Green prescribing—the prescribing of nature-based health interventions (iv) Plant-protein-based diets (v) Building energy analysis—although energy analysis is integral to decrease energy use within a clinic, the level of detail and engineering focus were not appropriate for our research team

Empirical research studies (qualitative, quantitative, mixed methods), thesis, and abstracts and conference proceedings, published in English between 1990 and June 2022, were included. Commentaries or opinion pieces which did not include a tool were excluded. Some topics, which could support sustainable healthcare practices were excluded if articles lacked a direct link with environmental outcomes (antibiotic stewardship and deprescribing), and/or where the literature on the topic is vast and extrapolating data was beyond the scope of the research team (all). On this basis we excluded: antibiotic stewardship, deprescribing, green-prescribing (prescribing of nature-based health interventions), and advice on plant protein-based diets.^19–21^ We also excluded studies focussing on building energy analysis as that was beyond our qualifications as a team.

### Search strategy

We searched 4 databases (PubMed, Embase, Scopus, and CINAHL) and 2 search engines (Google Scholar and Google). We completed a preliminary search on Google Scholar to identify search terms, worked with a medical librarian (Diane Lorenzetti) to develop a search string, which was then piloted and refined. The search strategies for each database and the search engine can be found in Supplementary Table A. Article tracking, review, and extraction were completed on Covidence (covidence.org). Duplicates were removed. Screening and full article review were completed by 2 or more independent reviewers (Sonja Wicklum, Kate Nuique, Jessica Zhang, and Colleen Nesbitt) with all disagreements concluded by a third reviewer after discussion. Manual searching on organizational websites about sustainable healthcare was also completed.

### Data extraction

Data extraction was completed on Covidence by independent reviewers (Sonja Wicklum, Kate Nuique, Jessica Zhang, and Colleen Nesbitt). Data extraction included author, title, publication source and date, target audience of article or aid, presence of educational materials (toolkits only), aim and description of articles presenting a singular tool/aid/project, and methods and results for articles describing interventions. We were unable to locate 1 article, despite assistance from our institutional librarian.

### Toolkit evaluation

All toolkits were evaluated separately. We acknowledge that assessment of article quality or risk of bias is not included in routine scoping reviews.^13^ In this circumstance, as our objective was to support family doctors integrating environmentally sustainable clinical care and operations, we deemed it important to provide basic evaluative elements for the reader. To the authors’ knowledge there is no formal critical appraisal tool for toolkits, therefore the evaluation was based on work by Yamada et al.,^22^ a systematic review of the effectiveness of toolkits as knowledge translation strategies. As per Yamada et al.,^22^ we applied the following appraisal criteria: (i) clear description of purpose; (ii) evidence-based elements; (iii) detailed implementation process; and (iv) rigorous evaluation plan combining outcome and process measures. In addition, we added a criterion to indicate if strategies to adapt to a community-based family practice setting were included.

## Results

**Fig. 1.**
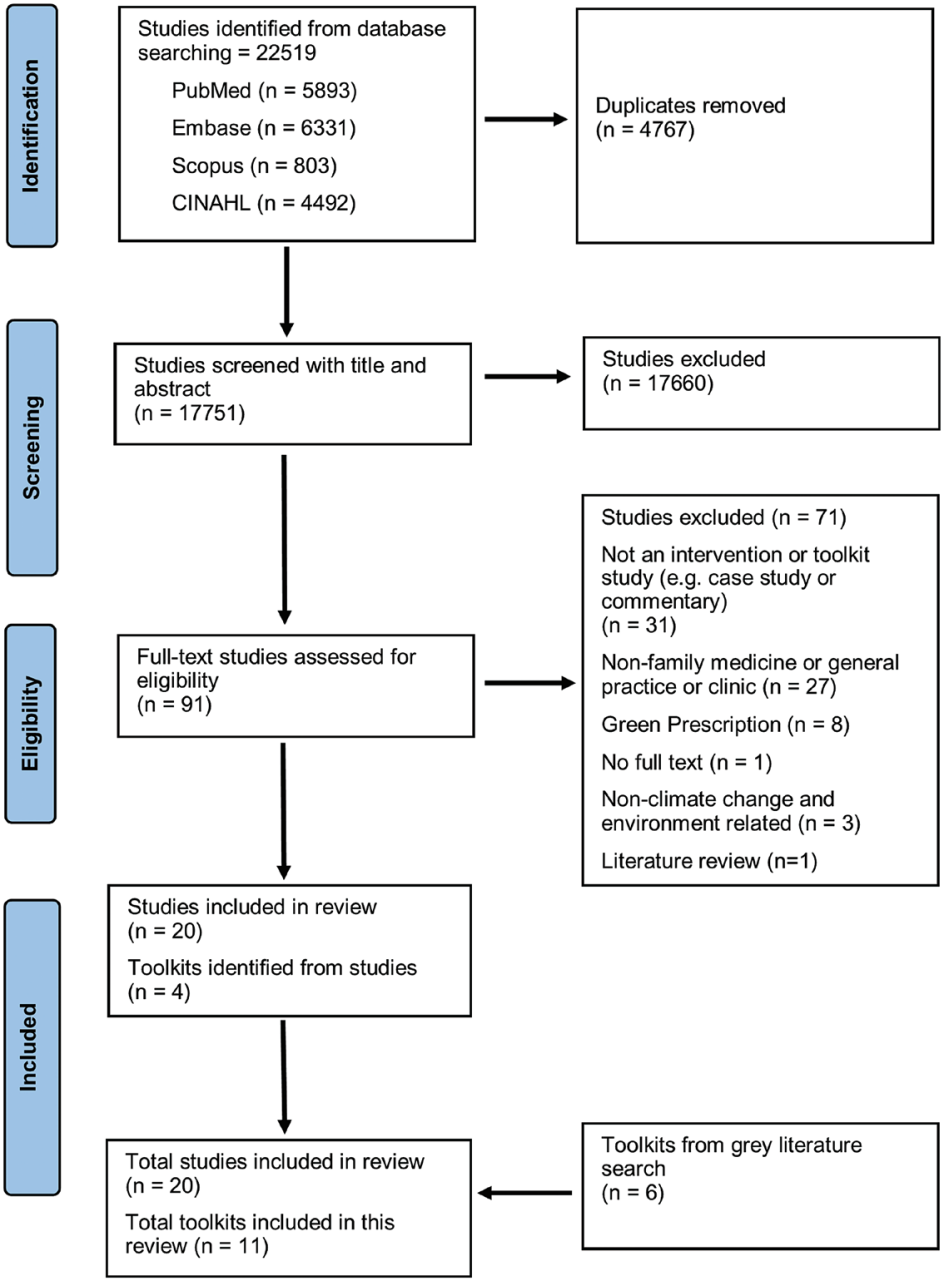
PRISMA flow diagram.

Our database search identified 17,751 articles after duplicates were removed. When assessing full texts for eligibility 4 articles were commentaries directing the reader to toolkits. These articles were not included in our review but the 4 toolkits they identified were. Complete screening of eligibility resulted in 20 relevant published articles that include an aid and 11 toolkits (Fig. 1). Most of the articles and toolkits originate in the Western hemisphere and Europe: 7 from the United Kingdom, 8 from the United States, 4 from Canada, and 1 each from the Americas, Brazil, and Switzerland. Only 1 article came from another continent (Africa).^23^ Many articles and toolkits focus on clinical operations, covering a broad scope of topics, as overviewed in Table 2. The clinical care areas that were addressed included active transportation,^9,12,24–39^ appropriate prescribing,^9,38–41^ social prescribing,^9,25,42^ mercury and lead as environmental contaminants, respiratory diseases, the value of connection of care providers to patients through community gardens, and medication disposal education.^9,12,29,37–41,43^

**Table 2.** Topics reviewed in articles and toolkits.

	Waste and recycling	Water and/or food	Energy, carbon, and greenhouse gases	Medical devices—single use	Built environment	Procurement of medical and office supplies	Anaesthetic gases	Medication disposal	Transport and exercise	Prescribing/deprescribing	Social prescribing^a^	Quality improvement projects outlined	Medical education components
*Articles*													
Babatunde^23^ 2018 Nigeria			✓			✓							
Barlow^44^ 2017 United States	✓		✓		✓	✓							✓
Blau^24^ 2016 Canada	✓	✓	✓	✓		✓			✓			✓	✓
Floss^45^ 2021 Brazil													✓
Fogarty^46^ 2008 Australia													✓
Gillam^25^ 2011 United Kingdom	✓					✓			✓		✓	✓	✓
Horton^26^ 2007 Australia	✓				✓				✓				
Kay^27^ 2020 Australia	✓	✓	✓		✓				✓				
Magzamen^47^2011 United States		✓			✓								
Murchie^28^ 2007 United Kingdom									✓				
Nichols^29^ 2011 United Kingdom	✓	✓	✓			✓		✓	✓				
Nicolet^30^ 2022 Switzerland	✓		✓	✓	✓	✓			✓				
Pedley^42^ 2019 United States		✓									✓		
Phipps^31^ 2011 New Zealand	✓	✓	✓	✓	✓	✓			✓				
Pistoll^32^ 2017 Australia									✓				
Last^40^ 2021 United States	✓		✓	✓		✓		✓		✓			
Tait^33^ 2018 Australia		✓	✓		✓				✓				
Ulrich^48^ 2010 United States	✓	✓	✓		✓							✓	
*Toolkits*													
Climate Change Toolkit^34^	✓	✓	✓	✓	✓	✓	✓		✓				
Climate Change Toolkit for Health Professionals^35^		✓	✓	✓	✓	✓	✓		✓				✓
GHG + H_2_O Green Facility Toolkit^36^		✓	✓		✓	✓			✓				
Green Impact for Health Toolkit^9^	✓	✓	✓	✓	✓	✓		✓	✓	✓	✓	✓	✓
Green Office Toolkit^37^	✓	✓	✓	✓		✓	✓	✓	✓				
Greener Practice Asthma Toolkit^41^	✓							✓		✓		✓	✓
Greening General Practice: A Toolkit for Sustainable Practice^38^	✓	✓	✓	✓	✓	✓		✓	✓	✓			
My Green Doctor^37^	✓	✓	✓	✓	✓	✓		✓	✓				
Practice Greenhealth^49^	✓	✓	✓	✓	✓	✓	✓		✓				✓
Smart Hospitals Toolkit^43^	✓	✓	✓		✓			✓					
The Greener Respiratory Pathway^39^	✓		✓					✓	✓	✓			

^a^Defined by Green Impact for Health as “healthcare practitioners advising and encouraging low carbon non-pharmaceutical interventions to patients affected by chronic health conditions, mental health problems, and social isolation.”

We start by describing the articles found in the scoping review and then present toolkits identified and their appraisal.

### Articles

Of the 20 articles found, 14 describe a single aid (Table 3) and 6 describe an intervention (Table 4).

**Table 3.** Articles reporting a single aid to support greening family medicine clinic operations and care.

Author	Knowledge user	Focus on clinic operations or clinical care	Aim	Description
Babatunde^23^ 2018 Nigeria	Clinic manager	Clinic operations	To introduce concept of “demand-side management (DSM)” and how it can lead to carbon footprint reduction, with a special focus on opportunities in rural health clinic settings	Chapter of book titled, “Environmental Carbon Footprints—Industrial Case Studies”
Barlow^44^ 2017 United States	Clinic manager	Clinic operations	To inform healthcare resource and material mangers on implementing initiatives addressing sustainability	Commentary with suggested checklist for management changes
Blau^24^ 2016 Canada	Primary care provider; specialist care provider; clinic manager	Clinic operations	To motivate and educate the need for greening healthcare and share a primer and checklist for greener family medicine clinics	Commentary with suggestions. Emphasizes need to ensure family medicine educational settings commit to “environment in everyday work” and proposes role of learners as conduits for change Promotes use of Sustainability Tracking, Assessment and Rating System of the Association for the Advancement of Sustainability in Higher Education
Gillam^25^ 2011 United Kingdom	Primary care provider	Clinic operations and clinical care	To explore what is needed in registrar (resident) training to prepare GPs for role in developing more sustainable healthcare system to protect population health	Commentary with suggested checklist for management and clinical care changes
Horton^26^ 2007 Australia	Primary care provider	Clinical care	To highlight areas that general practitioners (GPs) could engage with patients and the broader community to promote sustainable healthcare, addressing individual and environmental health simultaneously	Commentary with suggested clinical care changes
Magzamen^47^ 2011 United States	Specialist care provider	Clinic operations and clinical care	To review effects of 3 environmental contaminants and discuss ways paediatricians can act as individuals and through advocacy to impact these	Reviewed methylmercury, lead and asthma and discussed clinical care strategies to identify and support patients at risk. Also discussed advocacy as a means of supporting community level change to mitigate causal environmental toxins
Murchie^28^ 2007 United Kingdom	Primary care provider; clinic manager	Clinical care	To evaluate the environmental impact of a complex healthcare intervention and determine if evidence supports a primary care, GP follow-up model for melanoma	Partial carbon footprint analysis of clinical care pathway using life-cycle analysis tool. One year of GP follow-up versus hospital revealed a conservative reduction of 39 g CO_2 _in 1 year
Nicolet^30^ 2022 Switzerland	Primary care provider; clinic manager	Clinic operations	To quantify the average carbon footprint of a primary care consultation, describe differences between primary care practices (best, worst, and average performing) in western Switzerland and identify opportunities for mitigation	Average practice produced 30 tonnes of CO_2_/year, 45.7% from staff and patient transport, 29.8% for heating. Simulation of best and worst scenarios demonstrated a 10-fold difference
Phipps^31^ 2011 New Zealand	Primary care provider; clinic manager	Clinic operations	To provide an overview of climate change-related impacts in New Zealand and tips to green general practice	Summary of potential health impacts on climate change on New Zealand List of tips for greening general practice across several areas. Directs to Greening Your Practice Toolkit greeningyourpractice@gmail.com (see toolkits).
Podein^50^ 2010 United States	Primary care provider; specialist care provider; clinic manager	Clinic operations and clinical care	To introduce concepts of sustainability, give practice pearls and resources for physicians and clinic managers	Provides background and introduces resources. A resource is truncated, and a checklist created, based on Practice Greenhealth by Teleosis (see Table 2). Recommends footprintnetwork.org for individual carbon footprint analysis
Last^40^ 2021 United Kingdom	Primary care; allied team; clinic manager	Clinical care	To introduce environmentally conscious respiratory disease management to providers, with inclusion of environmental contributors, diagnosis, management, and medication choices	Description of strategies to reduce the environmental impact of respiratory disease management
Tait^33^ 2018 Australia	Primary care provider	Clinical care	To introduce GPs to heat-related morbidity and mortality and present a model to guide clinical care and prevention including primordial (societal level advocacy activity), primary and secondary prevention. Illustrated by a case study	Shows how GPs can act at all 3 levels of prevention
Ulrich^48^ 2010 United States	Primary care provider; clinic manager	Clinic operations	To discuss why doctors need to be conscious of the environment and offer suggestions for implementing eco-friendly behaviours into their medical practices	Commentary with suggested checklist for management changes
Walsh^51^ 2018 United Kingdom	Primary care provider; clinic manager	Clinic operations	To determine the environmental impact, carbon footprint, of completing e-learning for continuing professional development	High users of e-learning (>30 h/year, assuming 50 h/year average) can reduce their carbon footprint by 18.5 kg of CO_2_, through reduction of travel and relatively small amount of reduction from printing

**Table 4.** Articles describing interventions to promote sustainable healthcare.

Author	Target audience	Aim	Study design	Results
Floss^45^ 2021 Brazil	Primary care provider; specialist care provider; clinic manager	To describe course creation and development and assesses impact of the Planetary health Massive Open Online Course (MOOC).	Pre/post survey, analysis of “action plans”	Educational intervention. Of 2,777 enrolled, 1,237 consented to study, 49.8% completed course and 241 action plans were created. Deemed successful pilot with future iterations underway. Recognized need to better incorporate Indigenous perspectives. Required good connectivity, alternate resources need to be available to those without or with intermittent, connectivity.
Fogarty^46^ 2008 Australia	Primary care provider; clinic manager	To determine if an environmental educational program for general practitioners would lead to changes in sustainability within their practice.	Pre/post/3-month follow-up survey	Self-report surveys. Determined to be a successful pilot and expansion to GPs more widely may prove useful.
Kay^27^ 2020 Australia	Primary care provider; clinic manager	To investigate the effectiveness of a primary care health promotion project addressing environmental sustainability and equity in primary care partnerships.	Community-based participatory action study; qualitative focus groups and interviews	Thirty-two projects identified, topics covered: (i) caring for environment, access to nature and Indigenous participation, (ii) sustainability of housing, thermal comfort, and reducing energy costs, and (iii) sustainable and active transport. Decline in the number of partnerships that had environmental sustainability as a priority between 2009 and 2013. Did not evaluate the impacts or outcomes of the 32 projects.
Nichols^29^ 2011 United Kingdom	Primary care provider; clinic manager	To examine successful actions plan to reduce carbon emissions, as created and delivered by primary care trusts (PCTs)^a^.	Survey	50% response rate (7 of 14 directors of public health). 100% stated they had a sustainability strategy. Strategies included low-energy design, waste management, recycling, emissions, water, sourcing of food. “Patchy” evidence of successful implementation. Primary care trusts have since been abandoned.
Pedley^42^ 2019 United States	Primary care provider; clinic manager	To assess benefits of a community garden established by a medical clinic situated in a food desert.	Mixed—analysis of food production and engagement of staff, and Commentary	Garden produced 1,400–1,500 pounds of produce per year. Provided >3,000 opportunities for clinic personnel to interact with volunteers. Promoted green physical activity and working together. No measures of physical activity were measured.
Pistoll^32^ 2017 Australia	Primary care provider; clinic manager	To explore general practitioners’ (GPs’) ideas around active transport and its promotion.	Qualitative study	Ten semistructured interviews with GPs. Awareness of “active transport” as a term was limited but, without naming it, participants often discussed it with patients. Safety concerns limited its promotion.

#### Articles describing a single aid

Of the 14 articles, 7 focussed on clinic operations, 4 clinical care, and 3 addressed both areas. Most of articles reported simple checklists.^23–25,31,44,50^ Three articles focussed on carbon footprint analyses.^28,30,51^ This included demonstrating the environmental value of e-learning by reducing participants’ use of physical transport,^51^ the potential benefit of local GP follow-up over more distant hospital-based care^28^ and the variability of the carbon footprint in differing GP offices.^30^ One book chapter was included given its relevance to rural practitioners^23^ based in small-sized, rural hospitals. It introduced an approach to management called “demand-side management,” which advocated responding to a clearly defined need e.g. closing the clinic when not needed, or building a single room only when required.

#### Articles describing interventions to promote sustainable healthcare

Six articles described interventions to promote sustainable healthcare (Table 4), 4 of which described educational interventions.^24,25,46,45^ Educational interventions ranged from a large scale massive open online course (MOOC)^45^ to smaller educational initiatives^46^ and exploratory studies.^29,32^ The MOOC reached many individuals—midway through that project, the scope changed from practitioners to the public due to high demand. The MOOC included “action plan development” as part of the course, supporting implementation of change.

Other interventions evaluated the impact of “action plans” of a primary care and health promotion partnership^29^; the benefit of a medical clinic establishing a community garden^42^ and exploring GP awareness and promotion of active transport,^32^ such as walking or biking to complete daily activities in place of motorized transport. No study evaluated the implementation of integrating sustainable healthcare into practice.

### Toolkits

We identified 11 toolkits (Table 5). Three toolkits, Practice Greenhealth,^49^ The Smart Hospital Toolkit,^43^ and The GHG + H_2_O Green Facility Toolkit^36^ are primarily hospital focussed but include information about small- and medium-sized facilities and were included as they may inform rural family practices that operate in clinics attached to small-sized healthcare facilities.

**Table 5.** Background information and target audience of toolkits that support greening clinical care and clinic management for family doctors.

	Author/editor and/or association	Educational materials for patients	Knowledge user: primary care providers	Knowledge user: clinic managers
Climate Change Toolkit^34^ United States May 2016^a^ https://www.acponline.org/advocacy/advocacy-in-action/climate-change-toolkit	American College of Physicians	✓	✓	
Climate Change Toolkit for Health Professionals^35^ Canada April 2019^a^ https://cape.ca/blog-health-professionals/	Kim Perrotta, Executive Director Canadian Association for Physicians for the Environment (CAPE)	✓	✓	
GHG + H_2_O Green Facility Toolkit^36^ Canada n.d.^a^ https://greenhealthcare.ca/ghgwater/	The Canadian Coalition for Green Health Care		✓	✓
Green Impact for Health Toolkit^9^ United Kingdom 2014^a^ https://www.greenimpact.org.uk/GIforHealth/	Royal College of General Practitioners, Health Education England, the University of Bristol, and the National Union of Students Updated by general practice volunteers led by Terry Kemple	✓	✓	✓
Green Office Toolkit^37^ Canada n.d.^a^ https://greenhealthcare.ca/green-office-toolkit/	Neil Arya, Jean Zigby, Jasmine J. Mah, Lisa J. Jing Mu, Lyn Marshall, Linda Varangu, and Kent Waddington CAPE, the Canadian Coalition for Green Health Care, McMaster University Family Medicine, Synergie Santé Environnement, and Women’s College Hospital Environment Health Clinic		✓	✓
Greener Practice Asthma Toolkit^41^ United Kingdom October 2021 (version 3.3.2)^a^ https://www.greenerpractice.co.uk/high-quality-and-low-carbon-asthma-care/	Dr Aarti Bansal, Dr Sarah Wikeley, and Dr Tamsin Ellis Greener Practice	✓	✓	
Greening General Practice: A Toolkit for Sustainable Practice^38^ New Zealand 2016 (version 2) 2011 (version 1)^a^ https://www.rnzcgp.org.nz/RNZCGP/Im_a_member/Climate_change_and_greening_your_general_practice.aspx	Dr Rebecca Randerson and Dr Rochelle Philips. Updated Dr Randerson, Royal New Zealand College of General Practitioners	✓	✓	✓
My Green Doctor^12^ United States 2010^a^ https://mygreendoctor.org/	Dr Todd Sack (Editor) Florida Medical Association Managed by My Green Doctor Foundation	✓	✓	✓
Practice Greenhealth^49^ United States n.d.^b^ https://practicegreenhealth.org/	Their own nonprofit membership organization		✓	✓
Smart Hospitals Toolkit^43^ The Americas region December 2012^a^ https://iris.paho.org/handle/10665.2/34977	Ms. Marissa Da Breo-Latchma Pan American Health Organization			✓
The Greener Respiratory Pathway^39^ United Kingdom n.d.^a^ https://www.pcrs-uk.org/greener-respiratory-pathway	The Primary Care Respiratory Society	✓	✓	

^a^Accessed 11 August 2022.

^b^Accessed 7 October 2022.

The scope of material covered by toolkits ranged from several topic areas (Green Impact for Health and My Green Doctor)^9,12^ to those which focussed on 1 specific area, such as The Greener Practice Asthma Toolkit and The Greener Respiratory Pathway.^39,41^

All toolkits provided basic knowledge and explained terms commonly used in climate change, planetary health, or sustainable healthcare. Most tools included educational material for healthcare providers’ clinic staff, and 7 provided resources for patients (Table 5).

The focus of most toolkits was on clinical operations, spanning procurement of office supplies, energy use, and waste and recycling (Table 2). Clinical care topics were less commonly addressed and included prescribing and medication disposal. Three toolkits focussed on medical education, one describes medical curriculum and training program opportunities,^35^ a second requires trainees, member doctors, and/or members of the team to complete and submit proof of courses/training,^9^ and 1 included QI projects that trainees could implement.^41^

#### Toolkit appraisal

In relation to critical appraisal (Table 6) toolkits that include implementation and adaptation processes, and discussion around barriers are considered most effective. Two toolkits made suggestions for how to implement change. My Green Doctor^12^ recommended easing into change by adding 5 min to medical staff meetings until interest and skills increased sufficiently for greening projects to be undertaken. In contrast, Green Impact for Health^9^ presented a suite of topics, the clinic can choose a topic and identify the degree of change to target. However, participation is limited to doctors practicing in the United Kingdom (nonresidents can visit and utilize tools but cannot track or report). My Green Doctor,^12^ Green Impact for Health,^9^ Practice Greenhealth,^49^ and the Smart Hospitals Toolkit^43^ recommend monitoring and reporting on a number of items including energy, water, waste, and cost-savings, and provide tiered options that allow a clinic to adapt the approach best suited for their unique situation. Only the Green Impact for Health Toolkit^9^ has robust tracking, evaluating, and reporting information. To date, an estimated 37,700 kg of CO_2_ has been saved through the combined efforts of participating practices.^9^ At the time of writing, My Green Doctor and Green Impact for Health have large numbers of practices enrolled, and Practice Greenhealth, which focusses on hospital-based changes but supports Community Health Centers, required login and we were unable to obtain detailed information beyond that listed in Tables 2, 5, and 6.

**Table 6.** Critical appraisal of toolkits^a^ that support greening clinical care and clinic management for family doctors.

	Clear purpose	Evidence-based elements	Detailed implementation process	Adaptation to family practice	Evaluation component to study effectiveness and success	Updated regularly
Climate Change Toolkit^34^	Yes	Yes	No	Short list of greening “tips” within document, refers reader to My Green Doctor for detailed implementation.	No	No^b^
Climate Change Toolkit for Health Professionals^35^	Yes	Yes	No	Module 8 contains information on engaging patients, refers reader to Green Office Toolkit. Majority of focus on education of reader.	No	No
GHG + H_2_O Green Facility Toolkit^36^	Yes	Yes	Yes	Hospital focus, section for small- and medium-sized facilities refers to PAHO Smart Hospitals Toolkit above.	See Smart Hospitals Toolkit	No
Green Impact for Health Toolkit^9^	Yes	Yes	Yes	Easy to use, comprehensive toolkit that covers multiple topics, gives clear direction, of note >1,000 practices participating^c^. Activity logs and archives for individual practices on main site. Multilevelled approach to interventions so participation can be tailored.	Comprehensive, tracking, evaluation, and reporting methods, and awards for implementation.	Yes
Green Office Toolkit^37^	Yes	Yes	Yes	Specific to individual medical practices, comprehensive, 30 pages, no assets (infographics, posters, etc.).	Suggestions for tracking energy, water, waste	No
Greener Practice Asthma Toolkit^41^	Yes	Yes	Yes	Part of Greener Practice, UK-based primary care sustainability network, provides useful assets, QI project support, links to Center for Sustainable Healthcare^e^ for education.	Comprehensive quality improvement project support	Yes
Greening General Practice: A Toolkit for Sustainable Practice^38^	Yes	Yes	Yes	Specific to individual medical practices, comprehensive, 40 pages, no assets.		Yes
My Green Doctor^12^	Yes	Yes	Yes	Specific to individual medical practices, extensive resources, easy to implement by beginning with minimal time commitment during medical staff meetings, multilevelled approach to interventions so participation can be tailored.	Suggestions for tracking energy, water, waste, cost-savings	Yes
Practice Greenhealth^49^	Yes	Yes	^d^	Comprehensive resource directed at hospitals; special membership required for community health centres.	Resources for tracking and auditing	Yes
Smart Hospitals Toolkit^43^	Yes	Yes	Yes	Hospital focus, small- and medium-sized facilities operations, Pan-American Health Organisation (PAHO)/World Health Organisation’s disaster risk reduction programme.	Resources for tools and tracking, cost/benefit analysis	No
The Greener Respiratory Pathway^39^	Yes	Yes	No	Comprehensive resource directed at primary care, with links to many assets, created by Primary Care Respiratory Society.	Through links	No

^a^Criteria derived from article by Yamada et al.^22^

^b^Updated according to 17 October 2017 Facebook post.

^c^From https://www.youtube.com/watch?v=nNP5MUBrV7k October 2021, Accessed 22 August 2022.

^d^Centre for Sustainable Healthcare, https://sustainablehealthcare.org.uk/courses/sustainable-primary-care, Accessed 22 August 2022.

Overall, there was a lack of evaluation of toolkit efficacy in terms of benefits or outcomes. All toolkits are freely available but the functionality of some is limited by geographical location, or the inability to track progress. Few toolkits addressed barriers to implementing change. Only 5 toolkits^9,12,38,41,49^ are updated regularly.

## Discussion

Family doctors experience climate anxiety, as do their patients, and many want to change their personal behaviours at home and work, and support their patients to do the same, but are unsure of their role in addressing the topic.^52,53^ This scoping review was developed to support family doctors in greening their clinical care and operations, map the literature, evaluate the toolkits and aids, and identify gaps.

The literature identified covered a wide range of topics, with more emphasis placed on clinical operations than direct patient care. Resources ranged from singular tools (typically checklists) to toolkits. The singular tools that were checklists, or singular projects covering 1 topic, generally lacked information about implementation and did not explore barriers. Though lacking in detail and breadth of addressing the issues, they may prove useful for clinics with limited staff interest or resources (time or financial), or they may serve as an introduction to the concepts of sustainable healthcare that does not seem overwhelming, but rather can feel like an encouraging first step. Toolkits also varied in their scope; some focussed on specific areas such as respiratory disease,^40^ while others (Green Impact for Health,^9^ My Green Doctor^12^) provided in-depth and comprehensive information across a range of healthcare and environmental sustainability topics.

All the toolkits identified were free to use and accessible online, and their curation herein as a single resource we anticipate being beneficial for family physicians ready to transition to environmentally sustainable clinical care but without the time to identify resources to hand. Family physicians are well positioned to leverage trusting patient relationships to both educate and turn concern into action.^54^

However, our review also identified some shortcomings in the literature. Few of the articles or toolkits described provided guidance on how to implement change and address barriers to implementation. Another gap in the literature is around capacity for estimating carbon foot printing of various processes and interventions in healthcare. Carbon footprinting was introduced as an aid in several of the articles and toolkits, although we found no studies that looked at the influence of carbon footprinting on behaviour in the family practice setting, or how this intersects with larger governmental initiatives to reduce carbon footprints and meet international emission commitments. Identifying and quantifying carbon “hotspots” in healthcare and how they intersect with providing care in the context of cost effectiveness and social resources is complex, but necessary to understand the compromises that may need to be discussed to make our healthcare systems viable and sustainable. These studies support the notion that family doctors in community can have a significant impact on the carbon footprint of healthcare but have not yet been proven; this is a fruitful topic for future inquiry. Significantly, none of the resources (aids or toolkits) identified in our review have been evaluated for implementation or cost effectiveness, and we suggest integrating a robust evaluation process that includes environmental, social, and economic impacts (the “triple bottom line”) as balanced against outcomes for patients and populations to determine the sustainable value of implementation strategies.

### Limitations

Despite our best attempts to develop a robust search strategy, it is possible that we may have overlooked or omitted articles or toolkits, and this challenge is compounded by the wide range of terms used in relation to planetary health. Also, although we searched the grey literature it is possible that we failed to identify resources. Our review was limited to the English language and consequently the resources we found predominantly originated in Western countries, and it is possible that additional resources in different languages exist, particularly given the geographical and local health system focus of several of the resources we found. To complement our search, we contacted leading authors in the field for further suggestions of resources we may not have found and to discuss our findings.

## Conclusion

There have been several commentaries and “calls to action” to family doctors and primary care to make changes to their clinical operations and clinical care.^4,55–58^ Our scoping review highlights the beginnings of educational initiatives and interventions, and the availability of toolkits to support family doctors, clinic managers, and staff to implement evidence-based changes in both their clinic operations and clinical care. Despite knowledge and best intentions, doctors struggle to change practice behaviour.^52,59,60^ Toolkits that support change are a step in the right direction. Though limited, excellent toolkits exist to support family doctors and their clinics to provide more environmentally conscious and sustainable healthcare.

## Supplementary Material

cmad006_suppl_Supplementary_Table_A

cmad006_suppl_Supplementary_Checklist

## Data Availability

Not applicable.
