# Supplementary material for: Greening Family Medicine clinic operations and clinical care, where do we start? A scoping review of toolkits and aids
Source: Fam Pract. 2023 Feb 2;40(3):473–85. doi: 10.1093/fampra/cmad006 (PMC10231359; doi:10.1093/fampra/cmad006)
Supplement: cmad006_suppl_Supplementary_Table_A [file cmad006_suppl_supplementary_table_a.pdf]

Supplementary Table A. Search strategies for each database and search engine

Database	Search
PubMed	(("green*"[Title/Abstract] OR "carbon footprint"[Title/Abstract] OR "net zero"[Title/Abstract] OR "reduc*"[Title/Abstract] AND "prescription"[Title/Abstract] OR "deprescription"[Title/Abstract] OR "waste management"[Title/Abstract] OR "active"[Title/Abstract] OR "public transport"[Title/Abstract] OR "plant based diet"[Title/Abstract] OR "recycl*"[Title/Abstract] OR "greenhouse gas"[Title/Abstract] OR "sustainable"[Title/Abstract] OR "climate change"[Title/Abstract] OR "climate"[Title/Abstract] OR "environmental impact"[Title/Abstract] OR "inhaler"[Title/Abstract] OR "climate-smart"[Title/Abstract])) AND (("tool*"[Title/Abstract] OR "aid"[Title/Abstract] OR "guideline"[Title/Abstract] OR "resource*"[Title/Abstract] OR "mitigat*"[Title/Abstract] OR "adapt*"[Title/Abstract])) AND (("clinic"[Title/Abstract] OR "office"[Title/Abstract] OR"family medicine"[Title/Abstract] OR "family doctor"[Title/Abstract] OR "family practice"[Title/Abstract] OR "family physician"[Title/Abstract] OR "primary care"[Title/Abstract] OR "general practice"[Title/Abstract] OR "general practitioner"[Title/Abstract] OR "GPs"[Title/Abstract] OR "clinical practice" [Title/Abstract] OR "clinic management" [Title/Abstract] OR "patient medical home"[Title/Abstract] OR "medical facility"[Title/Abstract] OR "medical centre"[Title/Abstract])) Filters: English, from 1990/1/1 - 2022/6/30
Embase	1. green*.mp.<br>2. exp Carbon Footprint/<br>3. net zero.mp.<br>4. reduce prescription.mp.<br>5. exp deprescriptions/<br>6. waste management.mp.<br>7. plant based diet.mp.<br>8. exp Greenhouse Gases/<br>9. exp Climate/<br>10. exp Recycling/<br>11. environmental impact.mp.<br>12. sustainable.mp.<br>13. inhaler.mp.<br>14. climate smart.mp.<br>15. transport.mp.<br>16. 1 or 2 or 3 or 4 or 5 or 6 or 7 or 8 or 9 or 10 or 11 or 12 or 13 or 14 or 15<br>17. tool*.mp.<br>18. aid*.mp.<br>19. mitigat*.mp.<br>20. adapt*.mp.<br>21. resource*.mp.<br>22. guideline.mp.<br>23. 17 or 18 or 19 or 20 or 21 or 22<br>24. office*.mp.

25. family medicine.mp.
26. family doctor*.mp.
27. patient medical home.mp.
28. exp Family Practice/
29. exp Physicians, Family/
30. exp Primary Health Care/
31. exp general practitioner/
32. exp general practice/
33. clinic.mp.
34. exp primary medical care/
35. exp clinical practice/
36. clinic management.mp.
37. medical facility.mp.
38. medical clinic.mp.
39. 24 or 25 or 26 or 27 or 28 or 29 or 30 or 31 or 32 or 33 or 34 or 35 or 36 or 37 or 38
40. 16 and 23 and 39
41. limit 40 to (english language and yr="1990 - 2022")

Scopus

TITLE-ABS-KEY ("green*" OR "carbon footprint" OR "net zero" OR "reduc*" AND "prescription" OR "deprescription" OR "waste management" OR "active" OR "public transport" OR "plant based diet" OR "recycl*" OR "greenhouse gas" OR "sustainable" OR "climate change" OR "climate" OR "environmental impact" OR "inhaler" OR "climate-smart") AND TITLE-ABS-KEY ("tool*" OR "aid" OR "guideline" OR "resource*" OR "mitigat*" OR "adapt*") AND TITLE-ABS-KEY ("clinic" OR "office" OR "family medicine" OR "family doctor" OR "family practice" OR "family physician" OR "primary care" OR "general practice" OR "general practitioner" OR "gps" OR "clinical practice" OR "clinic management" OR "patient medical home" OR "medical facility" OR "medical centre") AND (LIMIT-TO (PUBYEAR , 2022) OR LIMIT-TO (PUBYEAR , 2021) OR LIMIT-TO (PUBYEAR , 2020) OR LIMIT-TO (PUBYEAR , 2019) OR LIMIT-TO (PUBYEAR , 2018) OR LIMIT-TO (PUBYEAR , 2017) OR LIMIT-TO (PUBYEAR , 2016) OR LIMIT-TO (PUBYEAR , 2015) OR LIMIT-TO (PUBYEAR , 2014) OR LIMIT-TO (PUBYEAR , 2013) OR LIMIT-TO (PUBYEAR , 2012) OR LIMIT-TO (PUBYEAR , 2011) OR LIMIT-TO (PUBYEAR , 2010) OR LIMIT-TO (PUBYEAR , 2009) OR LIMIT-TO (PUBYEAR , 2008) OR LIMIT-TO (PUBYEAR , 2007) OR LIMIT-TO (PUBYEAR , 2006) OR LIMIT-TO (PUBYEAR , 2005) OR LIMIT-TO (PUBYEAR , 2004) OR LIMIT-TO (PUBYEAR , 2003) OR LIMIT-TO (PUBYEAR , 2002) OR LIMIT-TO (PUBYEAR , 2001) OR LIMIT-TO (PUBYEAR , 2000) OR LIMIT-TO (PUBYEAR , 1999) OR LIMIT-TO (PUBYEAR , 1998) OR LIMIT-TO (PUBYEAR , 1997) OR LIMIT-TO (PUBYEAR , 1996) OR LIMIT-TO (PUBYEAR , 1995) OR LIMIT-TO (PUBYEAR , 1994) OR LIMIT-TO (PUBYEAR , 1993) OR LIMIT-TO (PUBYEAR , 1992) OR LIMIT-TO (PUBYEAR , 1991) OR LIMIT-TO (PUBYEAR , 1990)) AND (LIMIT-TO (LANGUAGE , "English")) AND (EXCLUDE (SUBJAREA , "AGRI") OR EXCLUDE (SUBJAREA , "IMMU") OR EXCLUDE (SUBJAREA , "BUSI") OR EXCLUDE (SUBJAREA , "MATH") OR EXCLUDE (SUBJAREA , "NEUR") OR EXCLUDE (SUBJAREA , "MATE") OR EXCLUDE (SUBJAREA , "MULT") OR EXCLUDE (SUBJAREA , "PSYC") OR EXCLUDE (SUBJAREA , "PHYS") OR EXCLUDE (SUBJAREA , "CENG") OR EXCLUDE (SUBJAREA , "ECON") OR EXCLUDE (SUBJAREA , "DECI")

	OR EXCLUDE (SUBJAREA , "ARTS") OR EXCLUDE (SUBJAREA , "DENT") OR EXCLUDE (SUBJAREA , "VETE") OR EXCLUDE (SUBJAREA , "Undefined")) AND (EXCLUDE (SUBJAREA , "COMP") OR EXCLUDE (SUBJAREA , "CHEM")) AND (EXCLUDE (SUBJAREA , "EART")) AND (EXCLUDE (SUBJAREA , "BIOC")) AND (EXCLUDE (SUBJAREA , "HEAL")) AND (EXCLUDE (SUBJAREA , "PHAR")) AND (EXCLUDE (SUBJAREA , "SOCI")) AND (EXCLUDE (SUBJAREA , "ENER"))
CINAHL	("green*" OR "carbon footprint" OR "net zero" OR "reduc*" AND "prescription" OR "deprescription" OR "waste management" OR "active" OR "public transport" OR "plant based diet" OR "recycl*" OR "greenhouse gas" OR "sustainable" OR "climate change" OR "climate" OR "environmental impact" OR "inhaler" OR "climate-smart") AND ("tool*" OR "aid" OR "guideline" OR "resource*" OR "mitigat*" OR "adapt*") AND ("clinic" OR "office" OR "family medicine" OR ...<br>Limiters - Published Date: 19900101-20220630; English Language; Language: English

Grey Literature

Database	Search
Google	green climate change clinic office toolkit resource<br>green climate change general practice family medicine toolkit resource<br>Sustainable climate-smart facility clinic adaptation mitigation<br>Sustainable medical facility resource
Google Scholar	("family medicine" OR "general practice") AND ("toolkit" OR "mitigation" OR "adaptation" OR "resource" OR "aid") AND ("climate change"), site: .org, site: .ca, site: .gov, site: .edu, site: .int, site: .com<br><br>1990-2022<br>("health facility" OR "medical clinic") AND ("toolkit" OR "mitigation" OR "adaptation" OR "resource" OR "aid") AND ("climate change"), site: .org, site: .ca, site: .gov, site: .edu, site: .int, site: .com<br><br>1990-2022
